# Transformation and regeneration of DNA polymerase Θ mutant rice plants

**DOI:** 10.1002/pld3.526

**Published:** 2023-09-05

**Authors:** Ayako Nishizawa‐Yokoi, Stanton B. Gelvin

**Affiliations:** ^1^ Institute of Agrobiological Sciences National Agriculture and Food Research Organization Tsukuba Japan; ^2^ Department of Biological Sciences Purdue University West Lafayette Indiana USA

**Keywords:** *Agrobacterium*‐mediated plant transformation, DNA polymerase theta, plant regeneration, rice

## Abstract

*Agrobacterium* T‐DNA integration into the plant genome is essential for the process of transgenesis and is widely used for genome engineering. The importance of the non‐homologous end‐joining (NHEJ) protein DNA polymerase Θ, encoded by the *PolQ* gene, for T‐DNA integration is controversial, with some groups claiming it is essential whereas others claim T‐DNA integration in *Arabidopsis* and rice *polQ* mutant plant tissue. Because of pleiotropic effects of PolQ loss on plant development, scientists have previously had difficulty regenerating transgenic *polQ* mutant plants. We describe a protocol for regenerating transgenic *polQ* mutant rice plants using a sequential transformation method. This protocol may be applicable to other plant species.

## 1 INTRODUCTION

T‐DNA (transferred DNA) integration into the host genome is the final step of *Agrobacterium*‐mediated transformation and is crucial for generating transgenic plants. The mechanism of T‐DNA integration most likely depends on host DNA repair enzymes, although a role for *Agrobacterium* VirD2 protein, which is covalently linked to the 5′ end of the single‐strand transferred DNA (T‐strands), has also been proposed (for review, see Gelvin, 2021). The participation of specific non‐homologous end‐joining (NHEJ) DNA repair proteins has been suggested, but the roles of specific proteins in T‐DNA integration are controversial: mutagenesis of some NHEJ genes, such as *Ku80* and *Lig4*, in *Arabidopsis*, and rice has resulted in decreased stable transformation in some studies (Friesner & Britt, 2003; Jia et al., 2012; Li et al., 2005; Mestiri et al., 2014; Saika et al., 2014) but either no change (Gallego et al., 2003; van Attikum et al., 2003) or increased (Park et al., 2015; Vaghchhipawala et al., 2012) stable transformation in other studies.

More recently, van Kregten et al. (2016) published that the NHEJ protein DNA polymerase theta, encoded by the *PolQ* gene, is required for stable transformation of *Arabidopsis thaliana*. Various properties of PolQ protein, including its use of microhomology for priming DNA synthesis and its propensity to “template switch” during DNA replication (Kent et al., 2016; Schimmel et al., 2017, 2019; Wyatt et al., 2016), offer a mechanism for the generation of several unusual sequence configurations at T‐DNA/plant DNA junctions. Template switching can account for the presence of “filler” DNA between integrated T‐DNA sequences and plant DNA and for major chromosomal rearrangements and translocations frequently generated during T‐DNA integration (Clark & Krysan, 2010; Curtis et al., 2009; Hu et al., 2017; Jupe et al., 2019; Kleinboelting et al., 2015; Lafleuriell et al., 2004; Majhi et al., 2014; Nacry et al., 1998; Ruprecht et al., 2014; Tax & Vernon, 2001). Filler DNA sequences can derive from within the T‐DNA region, from elsewhere on T‐DNA binary vectors, from other replicons in *Agrobacterium*, or from plant DNA, often but not exclusively from sequences near the plant DNA integration site (Kleinboelting et al., 2015; Nishizawa‐Yokoi et al., 2021; Singer et al., 2022). Additionally, PolQ frequently uses microhomology to anneal resected DNA sequences to recombination target sites (Schimmel et al., 2019); microhomology is often found between T‐DNA sequences and pre‐integration sequences in the host target DNA (Kleinboelting et al., 2015; Kralemann et al., 2022; Nishizawa‐Yokoi et al., 2021). The importance of PolQ for capturing and integrating the 3′ end of T‐strands has recently been described by Kralemann et al. (2022). In addition, van Tol et al. (2022) attempted to improve the frequency of homologous recombination‐mediated gene targeting (GT) by taking advantage of the decrease in T‐DNA integration in *Arabidopsis polQ* mutants. Although the loss of PolQ activity resulted in precise GT without additional T‐DNA integration or ectopic GT events, the GT frequency was very low in *polQ* mutant plants as compared to that of wild‐type plants.

Despite the proposed importance of DNA polymerase theta in T‐DNA integration, Nishizawa‐Yokoi et al. (2021) showed that PolQ is not essential, at least in somatic cells. In their studies, T‐DNA was readily integrated into both the *Arabidopsis* and rice genomes of *polQ* mutant cells. However, pleiotropic effects resulting from the loss of PolQ activity resulted in difficulty in recovering transgenic *polQ* mutant plants. These effects included slow growth of *polQ* mutant *Arabidopsis* calli and the inability for *polQ* mutant rice calli to regenerate plants (Nishizawa‐Yokoi et al., 2021). In addition, attempts to generate transgenic *polQ* mutant *Arabidopsis* by a flower dip transformation protocol were unsuccessful. However, if the incoming T‐DNA expressed a wild‐type *Arabidopsis PolQ* cDNA, stable transformation was restored (Nishizawa‐Yokoi et al., 2021). We subsequently showed that expression of a rice *PolQ* cDNA on an incoming T‐DNA could reverse the developmental defect of rice callus regeneration, permitting regeneration of *polQ* mutant rice calli (Gelvin, 2021). These results suggested a mechanism to generate transgenic plants from *polQ* mutant rice calli and to confirm that T‐DNA could integrate into the rice genome in the absence of PolQ activity, as described below.

## 2 MATERIALS AND METHODS

### 2.1 Plant materials

*Oryza sativa* L. cv Nipponbare and *polQ* mutant rice (Nipponbare background) generated using CRISPR/Cas9 (Nishizawa‐Yokoi et al., 2021) were used in this study. *polQ* Mutant Lines 5, 14, and 20 carried 31‐bp deletion, 1‐bp deletion, and 1‐bp insertion homozygous mutations at target sites of CRISPR/Cas9 in the 5th exon of the *OsPolQ* gene and had segregated out T‐DNA encoding the CRISPR vector system via self‐pollination. Plant genotypes were determined by CAPS analysis and DNA sequence analysis using primers listed in Table S1.

### 2.2 *Agrobacterium*‐mediated transformation

*Agrobacterium*‐mediated transformation was performed as previously reported (Saika et al., 2012). Nipponbare and *polQ* mutant rice calli were grown on N6D medium (Toki et al., 2006) at 33°C for 4 weeks and were infected with *Agrobacterium tumefaciens* EHA105 (Hood et al., 1993) harboring the T‐DNA monitoring vector 35Smini::ELuc+gfbsd2 (Saika et al., 2012) for the first round of transformation. After 3 days of co‐cultivation at 23°C on solidified 2 N6‐AS medium, the calli were washed and cultured on N6D medium containing 10 mg/L blasticidin S (Fujifilm Wako Pure Chemical Industries, Japan) and 25 mg/L meropenem (Fujifilm Wako Pure Chemical Industries, Japan). After a 4‐week selection period, genomic DNA was extracted from clonally propagated blasticidin‐resistant calli and subjected to Suppression PCR to analyze the T‐DNA/plant DNA junction sequence. We chose NB_Eluc#9, polQ#5_Eluc#13, and polQ#20_Eluc#18 and retransformed them using *A. tumefaciens* EHA105 harboring the T‐DNA binary vector pZN/OsPolQ vector (Gelvin, 2021), which harbors in the T‐DNA region an expression cassette for a *OsPolQ* cDNA (maize poly‐ubiquitin1 promoter::*OsPolQ* cDNA::*Arabidopsis* ribulose‐bisphosphate carboxylase small subunit terminator) and a neomycin phosphotransferase II expression cassette (rice actin 1 promoter::*nptII*::*Arabidopsis* polyA‐binding protein terminator). Transgenic calli were selected on N6D medium containing 35 mg/L G418 (geneticin; Nacalai tesque, Japan) and 25 mg/L meropenem for 4 weeks, and G418‐resistant calli were transferred to solidified regeneration medium ReIII (Toki et al., 2006) containing 25 mg/L meropenem. Regenerated plants were subjected to Suppression PCR and RT‐PCR analysis to confirm the presence of the junction from the first‐round of T‐DNA integration and *PolQ* expression from the endogenous mutant *polQ* gene and the wild‐type *PolQ* cDNA transgene.

### 2.3 Suppression PCR

Genomic DNA was extracted from clonally propagated blasticidin S‐resistant calli transformed with the T‐DNA monitoring vector using Agencourt ChloroPure (Beckman Coulter) according to the manufacturer's instructions. Suppression PCR was performed with adaptors and primers listed in Table S1 according to Nishizawa‐Yokoi et al. (2021). The junctions isolated by suppression PCR were confirmed by PCR using a T‐DNA‐specific primer paired with a primer specific to the plant genomic DNA near the insertion site.

### 2.4 Reverse transcriptase‐polymerase chain reaction (RT‐PCR) analysis

Total RNA was extracted from the shoots of regenerated plants of double‐transformed NB_Eluc#9_PolQ, polQ #5_Eluc#13_PolQ, and polQ#20_Eluc#18_PolQ using an RNeasy Plant Mini Kit (Qiagen). First‐strand cDNA was synthesized using ReverTra Ace (TOYOBO) with random primers. The cDNAs encoding the endogenous *polQ* gene and the *PolQ* transgene were amplified from the first‐strand cDNA by PCR using primers listed in Table S1.

### 2.5 DNA blot analysis

Genomic DNA was extracted from leaves of T0 and T1 polQ#20_Eluc#18_PolQ plants using a Nucleon Phytopure extraction kit (Cytiva) according to the manufacturer's protocol. Two‐microgram genomic DNA was digested with *Eco*RI and fractionated by electrophoresis through a 1.0% agarose gel. DNA blot analysis was performed according to the digoxigenin Application Manual (Roche Diagnostics). Specific DNA probes for the T‐DNA and plant genomic DNA near the integration site in polQ#20_Eluc#18_PolQ plants were synthesized with a PCR DIG probe synthesis kit (Roche Diagnostics) according to the manufacturer's protocol. Primer sequences are listed in Table S1.

## 3 RESULTS AND ANALYSIS

**FIGURE 1:**
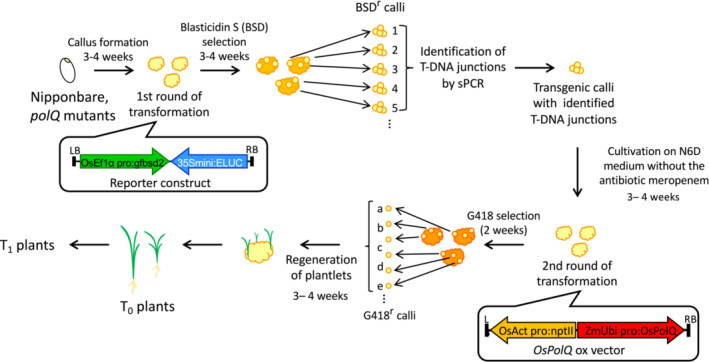
Experimental scheme to generate transgenic *polQ* mutant plants. Four‐week‐old Nipponbare, polQ#5, polQ#14, and polQ #20 rice calli were inoculated with *Agrobacterium* harboring T‐DNA with the reporter construct p35Smini:Eluc. Transformed calli were selected for 4 weeks on medium containing 10 mg/L Blasticidin S (BSD) and 25 mg/L meropenem. Genomic DNA was extracted from BSD‐resistant (BSD^r^) calli and subjected to suppression PCR (sPCR) to identify the T‐DNA junctions. Transgenic calli in which T‐DNA junctions were identified were transferred to medium lacking meropenem and cultured for 4 weeks. These calli were re‐infected with *Agrobacterium* harboring T‐DNA with the *OsPolQ* overexpression cassette, and plants were regenerated. Regenerated plants and their T_1_ progeny were subjected to segregation analysis of the p35Smini:Eluc T‐DNA and DNA blot analysis to confirm T‐DNA integration in the *polQ* mutant.

An outline of the experimental protocol is shown in Figure 1. Briefly, calli from wild‐type (Nipponbare) or *polQ* mutant rice lines were transformed by an *Agrobacterium* strain containing a binary vector with a GFP‐Blasticidin S (*Bsd*) fusion gene under the control of the rice elongation factor 1α promoter and a luciferase (*ELUC*) gene under the control of the CaMV35S minimal promoter in the T‐DNA (Saika et al., 2012). Blasticidin‐resistant calli were selected, and first‐round T‐DNA integration events were characterized. These transgenic calli were retransformed by an *Agrobacterium* strain containing a binary vector with a plant‐active *nptII* gene and a rice *PolQ* cDNA under the control of a maize ubiquitin (*Ubi*) promoter. G418‐resistant calli were selected, and T0 plants were regenerated. These double‐transformed plants set seed, T1 plants were germinated, and both the T0 and T1 plants were characterized for the presence of the first‐round T‐DNA and the presence of the original *polQ* mutant allele.

**FIGURE 2:**
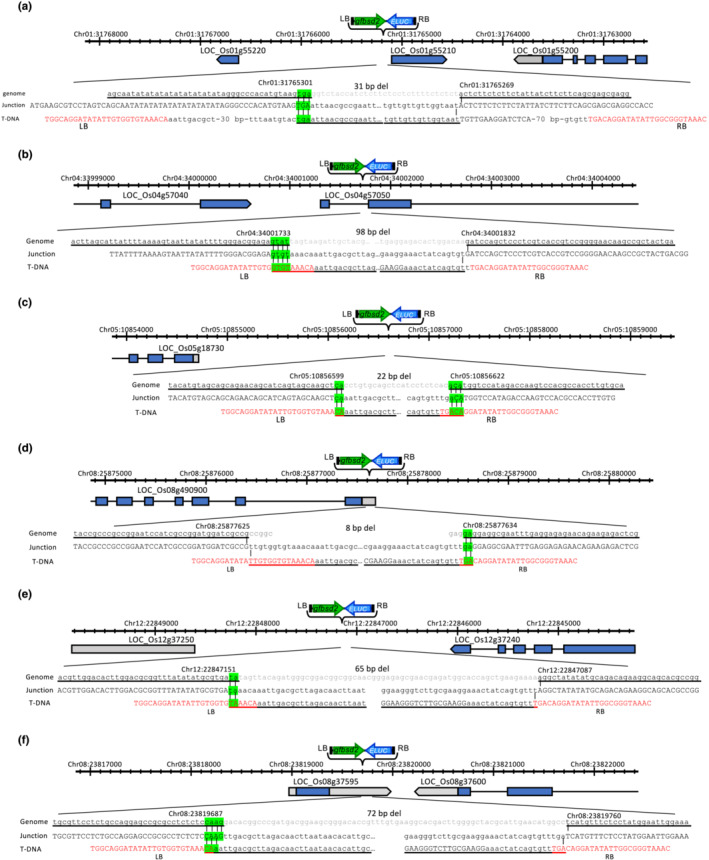
Schematic representation of T‐DNA integration sites and junction sequences in wild‐type (Nipponbare, NB), polQ#5, polQ#14, and polQ#20 mutant rice calli. The figure shows the T‐DNA integration sites into the genome in NB_Eluc#9 (a), polQ#5_Eluc#13 (b), polQ#14_Eluc#11 (c), polQ#14_Eluc#24 (d), polQ#20_Eluc#11 (e), and polQ#20_Eluc#18 (f). Gray letters indicate sequences deleted from the rice genome after T‐DNA integration. Red letters show the LB and RB sequences of T‐DNA. Green highlighted regions show regions of microhomology between T‐DNA and rice DNA.

We first transformed calli of wild‐type and three independent rice *polQ* mutants, *polQ#5*, *polQ#14*, and *polQ#20*. These mutants were generated by CRISPR disruption of an early exon in the rice *PolQ* gene (Nishizawa‐Yokoi et al., 2021). Because *polQ* mutant rice calli cannot readily regenerate into plants (Nishizawa‐Yokoi et al., 2021), we first characterized T‐DNA/plant DNA junctions isolated from blasticidin‐resistant calli. Figure 2 shows maps and sequences of some of these junction regions. The junctions resembled those previously characterized from transformed rice wild‐type and *polQ* mutant calli (Nishizawa‐Yokoi et al., 2021): Deletions within T‐DNA at left border (LB) regions are more extensive than at right border (RB) regions, deletions occur at the plant DNA target site, and microhomology between T‐DNA and the plant DNA target site can occur at both the LB and the RB. We chose integration events in wild‐type (NB_Eluc#9) and *polQ* mutant calli (polQ#5_Eluc#13 and polQ#20_Eluc#18) for further experimentation.

**FIGURE 3:**
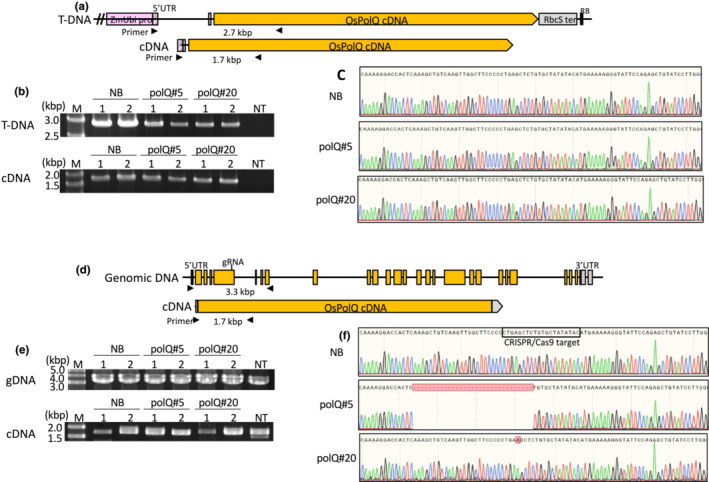
Analysis of transcripts from the *OsPolQ* overexpression construct and the endogenous *OsPolQ* gene in NB_Eluc#9_PolQ, polQ#5_Eluc#13_PolQ, and polQ#20_Eluc#18_PolQ regenerated plants. (a) Schematic of the T‐DNA (upper) and transcripts (lower) of the *OsPolQ* overexpression cassette; (b) PCR analysis of T‐DNA (upper) and cDNA (lower) with the primer set shown in (a) using genomic DNA and total RNA extracted from NB_Eluc#9_PolQ, polQ#5_Eluc#13_PolQ, and polQ#20_Eluc#18_PolQ regenerated plants, respectively; (c) DNA sequence analysis of cDNA derived from the *OsPolQ* overexpression cassette in NB_Eluc#9_PolQ (top), polQ#5_Eluc#13_PolQ (middle), and polQ#20_Eluc#18_PolQ (bottom); (d) schematic of the genomic DNA (upper) and transcripts (lower) of the endogenous *OsPolQ* gene in NB_Eluc#9_PolQ, polQ#5_Eluc#13_PolQ, and polQ#20_Eluc#18_PolQ regenerated plants; (e) PCR analysis of genomic DNA (upper) and cDNA (lower) with the primer set shown in (d) using genomic DNA and total RNA extracted from NB_Eluc#9_PolQ, polQ#5_Eluc#13_PolQ, and polQ#20_Eluc#18_PolQ regenerated plants, respectively; (f) DNA sequence analysis of cDNA derived from the endogenous *OsPolQ* gene in NB_Eluc#9_PolQ (top), polQ#5_Eluc#13_PolQ (middle), and polQ#20_Eluc#18_PolQ (bottom). These sequences are consistent with that of genomic DNA in NB, polQ#5, and polQ#20 mutants. cDNA, complementary DNA; gDNA, genomic DNA; NB, Nipponbare (wild‐type); NT, non‐transformed; pro, promoter; RB, T‐DNA right border; ter, terminator; Lanes 1 and 2 indicate independent biological replicates.

We retransformed NB_Eluc#9, polQ#5_Eluc#13, and polQ#20_Eluc#18 using an *Agrobacterium* strain containing within the T‐DNA a *nptII* gene and a wild‐type *PolQ* cDNA, selecting for G418‐resistant calli. Reverse transcription‐polymerase chain reaction (RT‐PCR) followed by DNA sequence analyses of the resulting amplicons showed that both the endogenous wild‐type *PolQ* gene, or mutant *polQ* gene, and the *PolQ* cDNA transgene were transcribed (Figure 3). Expression of the wild‐type *PolQ* cDNA permitted regeneration of polQ#5 and polQ#20 calli into plants. Regenerants from polQ#20 appeared phenotypically normal and set seed. However, regenerants from polQ#5 were dwarf and could not generate seeds, likely due to somaclonal mutations in this line.

**FIGURE 4:**
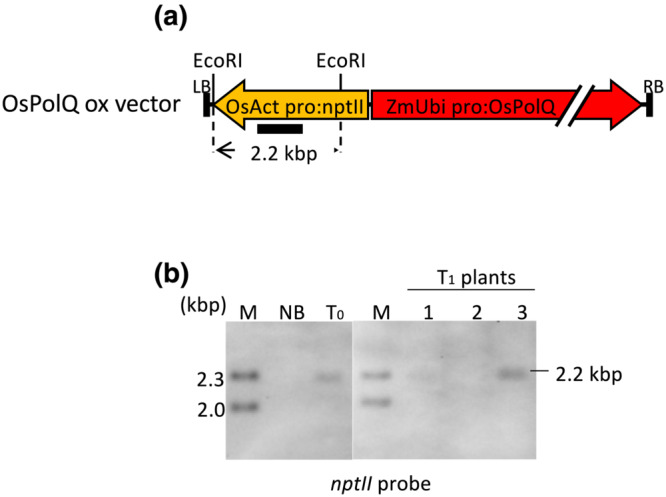
DNA blot segregation analysis of the OsPolQ overexpression (ox) vector in T_1_ progeny of the polQ#20_Eluc#18_PolQ line. (a) Schematic diagram of the OsPolQ ox vector showing the hybridization probe (bar below the map); (b) DNA blot analysis with the *nptII*‐specific probe shown in (a) using *Eco*RI‐digested genomic DNA of wild‐type (Nipponbare, NB), T_0_, and T_1_ plants of polQ#20_Eluc#18_PolQ.

**FIGURE 5:**
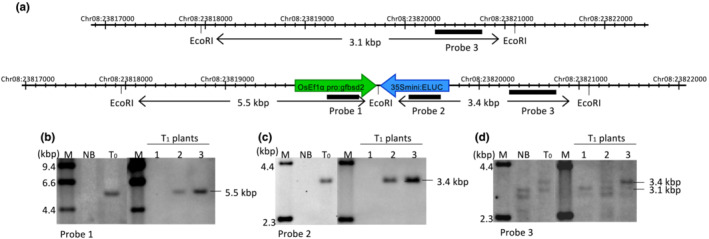
DNA blot segregation of p35Smini:Eluc in T_1_ progeny of the polQ#20_Eluc#18_PolQ line. (a) T‐DNA integration site in the polQ#20_Eluc#18_PolQ line. The lines indicate the genomic structure from Chr08:23817000 to Chr08:2382200 without (upper) or with (lower) p35Smini:Eluc integration in the polQ#20_Eluc#18_PolQ line, respectively; (b–d) DNA blot analysis using Probes 1 (b), 2 (c), or 3 (d) shown in (a) using *Eco*RI‐digested genomic DNA of wild‐type Nipponbare (NB), T_0_, and T_1_ plants of polQ#20_Eluc#18_PolQ. DNA blot analysis revealed that p35Smini:Eluc had segregated in the T_1_ progeny of polQ#20_Eluc#18_PolQ (Line 1, p35Smini:Eluc had segregated out; Lines 2 and 3, heterozygous and homozygous, respectively, for p35Smini:Eluc).

We further characterized DNA from polQ#20_Eluc#18 T0 and T1 plants by DNA blot analysis. Figure 4 shows that T‐DNA from the second transformation (containing the *nptII* and *PolQ* cDNA genes) was present in T0 plants and in self‐pollinated T1 Plants 1 and 3; however, this T‐DNA had segregated out in T1 Plant 2. Importantly, Figure 5 shows that T‐DNA from the initial transformation (containing the *GFP‐bsd* fusion and *Eluc* genes) was present in plants regenerated from the original *polQ* mutant calli and in T1 Plants 2 and 3; this initial T‐DNA had segregated out of T1 Plant 1. PCR analysis of three independent plants regenerated from polQ#20_Eluc#18 indicated that two of the three lines (a and c) segregated the *Eluc* gene in a Mendelian fashion (Table 1).

**TABLE 1:** Segregation ratio of the p35Smini:Eluc gene in the progeny of transgenic wild‐type (Nipponbare) and *polQ* mutant plants.

	Line of PolQ ox	No. of T1 plants analyzed	p35Smini:Eluc		
			−/−	+/−	+/+
Nipponbare x p35S mini: Eluc #9	a	16	4	9	3
polq#20 × p35S mini: Eluc #18	a	12	4	4	4
	b	15	3	9	3
	c	23	4	13	6

Abbreviations: −/−, null; +/−, heterozygous; +/+, homozygous for the p35Smini:Eluc transgene; ox, over‐expressing.

## 4 DISCUSSION

The role of plant NHEJ genes and proteins for T‐DNA integration has been controversial, with numerous studies purporting to show their importance for *Agrobacterium*‐mediated stable transformation (for review, see Gelvin, 2021). Only a few of these studies, however, have gone beyond selecting for stable transgenic plants and have conducted molecular analyses to confirm T‐DNA integration (Kralemann et al., 2022; Nishizawa‐Yokoi et al., 2021; Park et al., 2015; Vaghchhipawala et al., 2012). The importance of DNA polymerase theta (PolQ), a NHEJ protein, for T‐DNA integration in plant somatic cells has been disputed: van Kregten et al. (2016) and Kralemann et al. (2022) claimed that PolQ is required for efficient integration in *Arabidopsis* cells, whereas Nishizawa‐Yokoi et al. (2021) were able to detect T‐DNA integration into the genomes of both *Arabidopsis* and rice *polQ* mutants, at least in somatic cells. Both groups were unable to obtain stable transgenic plants through flower‐dip transformation of *Arabidopsis polQ* mutant plants.

The tebichi (“pig's feet”; cleft root) phenotype of *Arabidopsis polQ* mutant plants (Inagaki et al., 2006) frequently demonstrates incomplete penetrance that may depend on the degree of replicative environmental stress upon the plant (Nisa et al., 2021). We previously showed that, in addition to this phenotype, calli derived from roots of *Arabidopsis teb2* and *teb5* mutant plants proliferate slowly in culture, making the selection of *Agrobacterium*‐generated *polQ* mutant transgenic tissue difficult to obtain. We similarly showed that rice *polQ* mutant plants have a developmental phenotype: calli rarely regenerate plants under standard tissue culture regeneration conditions (Nishizawa‐Yokoi et al., 2021). These callus phenotypes likely derive from DNA damage accumulation during active cell proliferation. The PolQ‐mediated NHEJ pathway is often considered as a backup pathway for DNA ligase 4 (Lig4)‐mediated NHEJ. It has been reported that *polQ* mutant zebrafish cannot repair CRISPR/Cas‐induced or ionizing radiation‐induced DSBs, resulting in embryonic lethality or abnormal development (Thyme & Schier, 2016). Thus, PolQ‐mediated NHEJ is considered essential and dominant during the early development of a vertebrate. In germ cells of *Caenorhabditis elegans*, the PolQ‐mediated NHEJ pathway is essential for repair of CRISPR/Cas‐induced DSBs (van Schendel et al., 2015). Considering these findings, the PolQ‐mediated NHEJ pathway might be more prevalent in plants than is Lig4‐mediated NHEJ during some developmental stages or under some environmental conditions.

We have demonstrated that introduction of a wild‐type *PolQ* cDNA into *polQ* mutant *Arabidopsis* during flower‐dip transformation resulted in recovery of stably transformed seeds (Nishizawa‐Yokoi et al., 2021) and introduction of a wild‐type *PolQ* cDNA into *polQ* mutant rice calli allowed regeneration under standard tissue culture conditions (Gelvin, 2021). This latter result suggested a protocol to obtain stably transformed and regenerated rice plants initially harboring a *polQ* mutation: Calli derived from *polQ* mutant plants are first transformed by *Agrobacterium*, transgenic calli are selected, and T‐DNA integration is confirmed. A second round of transformation using an *Agrobacterium* strain harboring a wild‐type *PolQ* cDNA in the T‐DNA allows regeneration of the initial transformed *polQ* mutant plants.

It is important to suppress T‐DNA random integration into the host genome for the establishment of a transient transgene expression system from extrachromosomal T‐DNA or for optimization of homologous recombination‐mediated genome editing (gene targeting, GT) in plants. Although negative selection with genes encoding toxic proteins has been used to eliminate transgenic cells carrying T‐DNA randomly integrated into host cells, a large number of cells escape from negative selection by the integration of truncated T‐DNA (Nishizawa‐Yokoi et al., 2015; Terada et al., 2002). In human cells, dual loss of PolQ and Lig4 completely abolished the random integration of foreign DNA into the genome and exhibited 100% efficiency of GT (Saito et al., 2017). Likewise in plants, loss of both PolQ and Lig4 is a promising approach for the complete suppression of T‐DNA integration and improvement of GT efficiency. The protocol established in this study to obtain regenerated plants from *polQ* mutant plants would be required to rescue the GT cells from a *polQ/lig4* background.

In conclusion, T‐DNA can integrate into the genome of *polQ* mutant rice calli. T‐DNA from these transformants could be maintained and transmitted to the progeny of plants regenerated from these calli. These results confirm and extend our initial observations (Nishizawa‐Yokoi et al., 2021) that *polQ* mutant plants can integrate T‐DNA.

## AUTHOR CONTRIBUTIONS

Stanton B. Gelvin conceived the experiments, analyzed and interpreted the experimental results, and wrote the manuscript. Ayako Nishizawa‐Yokoi conducted the experiments, analyzed and interpreted the experimental results, and wrote the manuscript. The manuscript has been approved by both authors, who are accountable for all aspects of the work.

## CONFLICT OF INTEREST STATEMENT

The authors claim no conflict of interest, financial or otherwise, that might be perceived as influencing an author's objectivity.

## PEER REVIEW STATEMENT

The peer review history for this article is available in the supporting information for this article.

## DATA AVAILABILITY STATEMENT

The raw experimental data described are available upon request.

## Supporting information


**Supplemental Table S1.** Primers used in this study.

**Data S1** Peer review.
