# Supplementary material for: Transformation and regeneration of DNA polymerase Θ mutant rice plants
Source: Plant Direct. 2023 Sep 5;7(9):e526. doi: 10.1002/pld3.526 (PMC10480422; doi:10.1002/pld3.526)
Supplement: Supplementary file 1 — Supplemental Table S1. Primers used in this study. [file PLD3-7-e526-s002.docx]

Supplemental Table 1. Primers used in this study.

| Name | Sequence (5' to 3') | Purpose of use | Comments |
| --- | --- | --- | --- |
| AD-F | CTAATACGACTCACTATAGGGCTCGAGCGGCCGCCCGGGCAGGT | Adaptors used for Suppression PCR |  |
| AD-R | P-ACCTGCCC-NH2 |  | P, 5'-phosphorylation;  NH2, 3'-amination. |
| AP1 | GGATCCTAATACGACTCACTATAGGGC | Primary amplification of Suppression PCR (Pef1, for LB junction; ELUC, for RB junction) |  |
| Pef1α-1R sPCR | CGCAAACAAGGATAAGTGAGAAGATCTGAA |  |  |
| ELUC-1R sPCR | CTGATAGCCCTTGTATTTGATCAGCTCCTT |  |  |
| AP2 | AATAGGGCTCGAGCGGC | Nested amplification of Suppression PCR (Pef1, for LB junction; ELUC, for RB junction) |  |
| Pef1α-2R sPCR | TTGCGTTACGTTCAAGTCTCTGTAGGTTTC |  |  |
| ELUC-2R sPCR | CAGCAGGTTCTCTTCAGAGTCCAGTATGAT |  |  |
| p35SminiELUC LB Seq | CGTTACCCAACTTAATCGCCTTGCAG | Sequencing analysis of T-DNA junctions |  |
| ELUC-10R Seq | GCCGTACACCACGTTCTTCT |  |  |
| NBxELUC#9-RB | ctttaccaatcatttagggttcacg |  |  |
| NBxELUC#9-LB | cctcagataaacaccaaatgctacc |  |  |
| polq#5xELUC#13-LB | ccatagagtggaatttgatcgtctt |  |  |
| polq#5xELUC#13-RB | gtcacaagctaaacgagctacacaa |  |  |
| polq#14-11LB | aagagtgctcaatacggatgatagc |  |  |
| polq#14-11RB | cagatgttagcaacttaggggtgtt |  |  |
| polq#14-24LB | ctacattgctaactggctggagagt |  |  |
| polq#14-24RB | acaatcagagccaaaagcaatttag |  |  |
| polq#20-11LB | aacgagcagtacgactgaactaacc |  |  |
| polq#20-11RB | acctgaccacacacaaggaatttat |  |  |
| polq#20xELUC#18-LB | cggctagtgattgcatactgaattt |  |  |
| polq#20xELUC#18-RB | tgtcaaattgtgttgcaaaagttct |  |  |
| OsPolQ CAPS-F | AGAGTGGGTGACAGTGGTTCTT | CAPS analysis for the identification of *ospolq* mutant |  |
| OsPolQ CAPS-R | AAAAGGTGGAAGGTTGTGATTG |  |  |
| OsPolQ Forward1 | CATGTAAACAGCTCTCCGTTGCCTGTGA | Analysis of OsPolQ transcripts (OsPolQ Forward1 and OsPolQ exon7-R for endogenous PolQ transcripts; ZmUbicDNA-F and OsPolQ exon7-R for transgene) |  |
| OsPolQ exon7-R | CAGCACATATTGACACATAGGGAAG |  |  |
| ZmUbi cDNA-F | CGCTTCAAGGTCGACTCTAGA |  |  |
