# Supplementary material for: Transformation and regeneration of DNA polymerase Θ mutant rice plants
Source: Plant Direct. 2023 Sep 5;7(9):e526. doi: 10.1002/pld3.526 (PMC10480422; doi:10.1002/pld3.526)
Supplement: Supplementary file 2 — Data S1 Peer review. [file PLD3-7-e526-s001.docx]

**DECISION LETTERS – Round 1**

July 26, 2023

Dr. Stanton B Gelvin
Purdue University
West Lafayette, Indiana


RE: Transformation and regeneration of DNA polymerase theta mutant rice plants

Dear Dr. Gelvin:

Thank you for submitting to Plant Direct. All required reviews have been returned and we have now finished our evaluation of your manuscript. In light of the reviewers' comments, I anticipate accepting the manuscript for publication in Plant Direct, but I would like to give you an opportunity to incorporate some helpful suggestions from reviewer 2 regarding figure clarity (see notes below and attached file).

When uploading the revised version of this article, please be sure to include the following:

-A word document that contains your response to the reviewers. You should respond to each reviewer comment and note the changes made to the manuscript. If you do not agree with a reviewer's comment and choose not to make a suggested revision, please explain why. Please try to provide as complete an answer as possible to each reviewer's criticisms.
-A tracked changes document with each change highlighted
- A clean version of the latest version of the manuscript


To upload your revision, please click the link below.
https://plantdirect.msubmit.net/cgi-bin/main.plex?el=A2Lr7bZ4A5oIK1I7A9ftd7FtuRknuzn7by0wggCCkQZ

In order to provide as timely a service as possible, we ask that your revision is resubmitted within three months after receipt of this request. If an extension is needed, please send a request, along with a brief explanation, to the editorial office at plantdirect@wiley.com .

Please note that, in addition to publishing reviewer comments, the author's responses to review comments will also be published alongside the final version of the paper. If you would not like the author's responses to be published, please contact the editorial office at plantdirect@wiley.com .

Thank you very much for giving us an opportunity to review your work. I look forward to receiving the next version.
Sincerely,

Rebecca Mosher

Hsou-min Li

Editor, Plant Direct


---------------------------------------------------------------------------- Reviewer comments:
Reviewer #2:

Notes to authors:
In the Figure 3 legend, please define NT and explain what 1 and 2 refer to.
Panel F of Figure 3 would be much easier to interpret if there were a horizontal space between each sequence trace. As presented, it is not intuitively obvious that the single letter code refers to the trace below, rather than the one above, the letters. This problem is exacerbated by the red bar denoting the deletion in polQ#5; the placement of this bar directly below the sequence trace for NB makes it appear that the bar relates in some way to the top trace.
There are a few typos:
Line 16, Corresponding needs a "d"
Line 21, claiming should be claim
Line 135, weres should be were
Line 377, a comma is needed after "(D)"

**DECISION LETTERS – Round 2**

August 9, 2023
Dr. Stanton B Gelvin
Purdue University
West Lafayette, Indiana


MSID: 2023-01224R1
MS TITLE: Transformation and regeneration of DNA polymerase theta mutant rice plants

Dear Dr. Stanton Gelvin:

I am pleased to inform you that your manuscript "Transformation and regeneration of DNA polymerase theta mutant rice plants" has been accepted for publication in Plant Direct.

Your article will appear online in the next available issue of Plant Direct. To ensure your article gets published as quickly as possible, please pay attention to the steps detailed below. We have found that most of the delays happen at this stage, especially at the payment stage, so please respond as quickly as possible when prompted.

License Agreement: Once your article has been accepted it will move to Production and undergo admin and file checking - you may receive an email with any queries we have at this stage. When all required items are received by the publisher and queries resolved, the corresponding author will receive an email from Wiley's Author Services system which will ask them to log on at https://authorservices.wiley.com/bauthor and will present them with the appropriate license for completion. Your article cannot be published until both the signed license agreement and payment of the article fee have been received.

Payment of the Open Access Article Publication Fee: All articles published in Plant Direct are fully open access: immediately and freely available to read, download and share. Plant Direct charges a publication fee to cover the publication costs. The corresponding author for this manuscript should have already received a quote from the payments team (cs-openaccess@wiley.com) with the estimated article publication fee; please email cs-openaccess@wiley.com if this has not been received. The corresponding author should log on to the Wiley Author Services site, where the publication fee can be paid by credit card or an invoice. Pro Forma can also be requested. Payment of the publication charge must be received before the article will be published online.

Proofs: You will have the opportunity to look over your paper once more when you receive the author proofs for your article. The proofs will be with you in approximately two weeks. Please note that, in addition to publishing reviewer comments, the author's responses to review comments will also be published alongside the final version of the paper. If you would not like the author's responses to be published, please contact the editorial office at plantdirect@wiley.com .

Promotion of your article: You can help your research get the attention it deserves! Check out Wiley's free Promotion Guide for best-practice recommendations for promoting your work at www.wileyauthors.com/eeo/guide. And learn more about Wiley Editing Services which offers professional video, design, and writing services to create shareable video abstracts, infographics, conference posters, lay summaries, and research news stories for your research at www.wileyauthors.com/eeo/promotion.

Thank you again for your contribution to Plant Direct. If you have any questions, feel free to contact the editorial office at plantdirect@wiley.com .

Sincerely,

Rebecca Mosher

Hsou-min Li

Editor, Plant Direct


---------------------------------------------------------------------------- Editor comments


---------------------------------------------------------------------------- Reviewer comments:

**AUTHORS’ RESPONSE**

Dear Editor,

Thank you and Reviewer #2 (we did not receive any comments from Reviewer #1) for the very careful reading and useful comments on the manuscript. We have incorporated all the suggestions in this revised version:

In the Figure 3 legend, please define NT and explain what 1 and 2 refer to.

NT=non-transformed; 1 and 2 indicate independent biological replicates. We have now indicated these in the figure legend.

Panel F of Figure 3 would be much easier to interpret if there were a horizontal space between

each sequence trace. As presented, it is not intuitively obvious that the single letter code refers

to the trace below, rather than the one above, the letters. This problem is exacerbated by the

red bar denoting the deletion in polQ#5; the placement of this bar directly below the sequence

trace for NB makes it appear that the bar relates in some way to the top trace.

Thank you for your suggestion. We have revised Panels C and F of the figure accordingly.

There are a few typos:

Line 16, Corresponding needs a "d"

Line 21, claiming should be claim

Line 135, weres should be were

Line 377, a comma is needed after "(D)"

We have made all the suggested changes.

We hope that this revised manuscript is now suitable for publication in Plant Direct.

Sincerely,

Stan Gelvin (for both authors)
